# Seedling tolerance to cotyledon removal varies with seed size: A case of five legume species

**DOI:** 10.1002/ece3.3169

**Published:** 2017-06-22

**Authors:** Xiao Wen Hu, Rui Zhang, Yan Pei Wu, Carol C. Baskin

**Affiliations:** ^1^ State Key Laboratory of Grassland Agro‐ecosystems College of Pastoral Agriculture Science and Technology Lanzhou University Lanzhou China; ^2^ Department of Biology University of Kentucky Lexington KY USA; ^3^ Department of Plant and Soil Sciences University of Kentucky Lexington KY USA

**Keywords:** cotyledon damage, relative growth rate, seed mass, seedling survival

## Abstract

It is generally accepted that seedlings from large seeds are more tolerant to defoliation than those from small seeds due to the additional metabolic reserves present in the large seeds. However, information on the effects of amount of seed reserves (cotyledon removal) from seedlings resulting from large vs. small seeds on seedling growth and long‐term survival in the field is limited. Five legume species with different sizes of seeds were sown in the field and none, one, or both cotyledons removed 7 days after seedling emergence. Seedling biomass, relative growth rate (RGR) and survival were determined at different time. Cotyledon removal, species, and their interaction had significant effects on seedling growth and survival. During the period between 33 and 70 days, seedlings from large seeds had a significantly lower RGR than those from small seeds. Biomass, RGR, and survival of seedlings from large seeds were significantly reduced by removal one or both cotyledons, whereas those of seedlings from small seeds were not affected. Seed energy reserves are more important for the early growth of seedlings from large seeds than for those from small seeds. The overall effect of cotyledon removal on growth and survival varies with seed size (i.e., energy reserves) with seedlings from small seeds being less sensitive than those from large seeds under field conditions.

## 1 INTRODUCTION

Seed size varies from 0.0001 mg for the orchids to 20 kg for the double coconut (Harper & Moore, 1970; Moles et al., 2005), and these variations in mass have been assumed to have substantial effects on seed germination, seedling recruitment, and consequently plant fitness (Armstrong & Westoby, 1993; Hanley & May, 2006; Leishman, Wright, Moles, Westoby, & Fenner, 2000). Some studies have found that seedlings from large seeds are more successful than those from small seeds when they experience competition (Gross, 1984), defoliation (Armstrong & Westoby, 1993), shade (Leishman et al., 2000), or moisture stress (Baker, 1972; Leishman & Westoby, 1994). However, previous studies have also found that the advantage of large seeds decreased as seedling grew, particularly in resource‐rich environments, but not in unfavorable environments in either nursery (Seiwa & Kikuzawa, 1991) or field conditions (Seiwa & Kikuzawa, 1996).

Seedlings from large seeds are better able to cope with cotyledon removal or defoliation during early development than those from small seeds, evidenced in many species (Dalling & Aizprua, 1997; Dalling & Harms, 1999; Giertych & Suszka, 2011; Yi, Rachel, Bartlow, Agosta, & Steele, 2013; Yi, Wang, Liu, Liu, & Zhang, 2015; Yi et al., 2012). This difference may result because the additional metabolic reserves present in large seeds could buffer carbon losses (Dalling & Harms, 1999; Kitajima, 1996). However, seedlings from small seeds generally grow faster than those from large seeds, and thus, they may overcome the initial size advantage associated with large seed size (Baraloto, Forget, & Goldberg, 2005; Paz & Martínez‐Ramos, 2003; Rose & Poorter, 2003). Milberg and Lamont (1997) showed that seedlings of the small‐seeded species, *Eucalyptus loxophleba*, were less affected by removal of the cotyledons than those of the large‐seeded species, *E. todtiana* and *Hakea psilorrhyncha*. Hanley and May (2006) found that plant growth during the establishment phase was significantly reduced by cotyledon removal in six of nine species, but the two with the smallest seeds were not affected by cotyledon removal. Recent studies (Giertych & Suszka, 2011; Yi et al., 2012, 2013, 2015) on oaks (*Quercus*) showed that seed germination and seedling survival were little affected by cotyledon removal, suggesting that cotyledonary reserves in acorns may be not crucial for supporting seedling development and may serve as food for manipulating seed predators and dispersers.

These controversial results of seed size effects on seedling tolerance to cotyledon removal may be related to cotyledon type (Baraloto et al., 2005), seedling growth stage (Hanley & May, 2006; Seiwa & Kikuzawa, 1991, 1996), cotyledon removal time (Hanley & Fegan, 2007), and seedling growing conditions (Lamont & Groom, 2002). All of these factors may potentially confound the effects of seed mass and complicate the interpretation of results from cotyledon removal experiments. Moreover, in most cases, the effects of cotyledon removal on seedling growth and survival were determined in an artificially controlled environment with less stressful growth conditions than in the field. In fact, Hanley and May (2006) suggested that the results of their experiment on the effects of cotyledon damage at the seedling stage on plant growth and flowering might have been different if it had been conducted under field conditions. Thus, to our knowledge, the effects of both seed size and cotyledon removal on seedling growth and long‐term survival have not been tested under field conditions.

In this study, we used five epigeal legume species with seeds varying in size and determined the effect of cotyledon removal and seed size on seedling growth and survival at different growth stages under field conditions. We hypothesized that: (1) seedlings from large seeds are more dependent on seed reserves than those from small seeds, and thus, growth of seedlings from small seeds is less affected by cotyledon removal than that of seedlings from large seeds; (2) seedlings from small seeds have a higher relative growth rate than those from large seeds; thus, the initial advantage of seedling size from large seeds will decrease with seedling growth; (3) survival percentage of seedlings from large seeds is higher than that from small seeds at an early time but not in the long term.

## 2 MATERIALS AND METHODS

### 2.1 Seed collection

*Ammopiptanthus mongolicus* (Kom.) S.H. Cheng., *Lespedeza potaninii* Vass., *L*. *dahuric* (Laxm.) Schindl., *Melilotus albus* Desr., and *Sophora alopecuroides* L. are five common legume species in temperate zone arid and semi‐arid ecosystems, their seeds all have epigeal foliar cotyledons and thousand seed mass ranges from 1.6 g to 46.4 g. For these five species, insect predation at early seedling stages is very common in the field (Liu, 1998; personal observation), and thus, cotyledon removal may have effects on seedling performance and recruitment.

Seeds of *Ammopiptanthus mongolicus*,* Sophora alopecuroides,* and *Lespedeza potaninii* were collected from the Alax Desert, Inner Mongolia, China (105°34′ E, 39°05′ N; 1,360 m a.s.l.) in July, September and November 2012, respectively. Seeds of *Melilotus albus* and *L*. *dahurica* were collected from a field on the Yuzhong Campus of Lanzhou University (35°57′ N, 104°10′E, 1,700 m a.s.l.), Gansu Province, China, in July and November 2012, respectively. For each species, ripe pods were collected from more than 30 individual plants and taken to the laboratory, where seeds were removed from the pods, cleaned, and then stored dry in a paper bag at room conditions (RH 20–45%; 18–25°C) until used in experiments in 2013. The thousand seed mass of *Ammopiptanthus mongolicus*,* Sophora alopecuroides*,* Lespedeza potaninii*,* L*. *dahurica,* and *Melilotus albus* was 46.4 ± 0.37, 24.0 ± 0.35, 2.3 ± 0.10, 2.2 ± 0.16, and 1.6 ± 0.02 g (mean ± SE, *n* = 8), respectively.

### 2.2 Effect of cotyledon removal on seedling biomass and growth rate

This experiment was conducted at the Yuzhong campus (35°57′N, 104°10′E), Lanzhou University, Gansu Province from 18 May 2013 to 6 April 2014. For each species, seeds were sown in soil in 90 open‐ended PVC pots (15 cm diameter, 11 cm height) buried in the field with the rim 5 cm above the soil surface. Soil level in the pot was even with the soil surface. The soil placed in the pots was a mixture of silt (66.9%), clay (20.8%), and sand (12.3%), and available nitrogen, phosphate, and potassium was 12.1, 25.5, and 113.3 mg/kg, respectively.

To ensure uniform seedling emergence, seeds were presoaked in distilled water for 24 hr at 20°C before sowing. Five imbibed seeds were sown in each pot, and spray irrigation was applied twice to all the pots in the first week to ensure seedling emergence, and then, no irrigation was applied during the remainder of the experiment. Three days after sowing, most seedlings had emerged (21 May 2013). Two seedlings of similar size were kept in each pot, and the others were removed carefully by hand. Although there was a potential for competition between the two seedlings in each pot, this effect on the results was negligible based on the following reasons. (1) The bottom of the PVC pot was open; thus, root growth was not restricted by the size of the pot. (2) The diameter of the pot aboveground was 15 cm, providing space between the seedlings. (3) The pots were 50 cm apart; thus, there was no completion from plants in neighboring pots.

Seven days after seedling emergence (28 May 2013), plants of each species were divided into three equal groups (i.e., 30 replicate pots and 60 plants per treatment): 0% (control, cotyledons intact), 100%, or 50% cotyledon removal. In the 100% treatment, both cotyledons were removed from each seedling, while in the 50% treatment one of the cotyledons was removed. All cotyledons were removed at the node, using scissors. For each of the three groups, 10 seedlings each were harvested to determine total biomass at the beginning of treatment (28 May 2013) and after growth for 33 and 70 days (30 June and 6 August 2013, respectively). When seedlings were harvested 0, 33, and 70 days after treatment, one seedling was removed from each of 10 pots in each of the three groups. The second seedling in each of the 10 pots was used to determine survival. Dry mass was determined after drying seedlings at 80°C for 2 days. Seedling relative growth rate (RGR_1_ and RGR_2_) was calculated as follows:

$${\text{RGR}}_{1}=\left[\text{ln}\;\left({\text{DM}}_{1}\right)-\text{ln}\left({\text{DM}}_{0}\right)\right]/t_{1}$$

$${\text{RGR}}_{2}=\left[\text{ln}\;\left({\text{DM}}_{2}\right)-\text{ln}\;\left({\text{DM}}_{1}\right)\right]/t_{2},$$

where DM_0_, DM_1_, and DM_2_ correspond to dry biomass at the harvest of 28 May, 30 June, 6 August, respectively, and *t*_1_ and *t*_2_, time from 28 May to 30 June, and 30 June to 6 August, which is 33 days and 38 days, respectively.

### 2.3 Effect of cotyledon removal on seedling survival

Thirty seedlings for each group were used to determine percentages of survival. The percentage of survival (based on initial seedling number) was determined on 30 June 2013, 6 August 2013, and 10 April 2014. Total rainfall from 18 May 2013 to 30 June, 6 August 2013, and 10 April 2014 was 57.1, 221.3, and 414.4 mm, respectively. The site has an average annual temperature of 6.7°C with a minimum monthly mean of −5°C in January, and a maximum monthly mean of 23°C in July (Yuzhong weather station). Thus, seedlings were grown under natural light, temperature, and rainfall conditions.

### 2.4 Statistical analysis

Three‐way analysis of variance (ANOVA) was used to test the effects of species, time, and cotyledon removal on seedling biomass and relative growth rate, and mean comparison among treatments for each species were conducted with Duncan's multiple range test. To meet the assumptions of normality and homogeneity of ANOVA, seedling biomass was log transformed before analysis. Chi‐square test was used to test the effect of cotyledon removal on seedling survival of each growth stage. Pearson's correlation analysis was conducted to test the relationship between seed mass (log transformed) and seedling biomass, and seedling relative growth rate for each cotyledon removal treatment and time.

## 3 RESULTS

### 3.1 Effect of cotyledon removal on seedling biomass and relative growth rate

**Figure 1:**
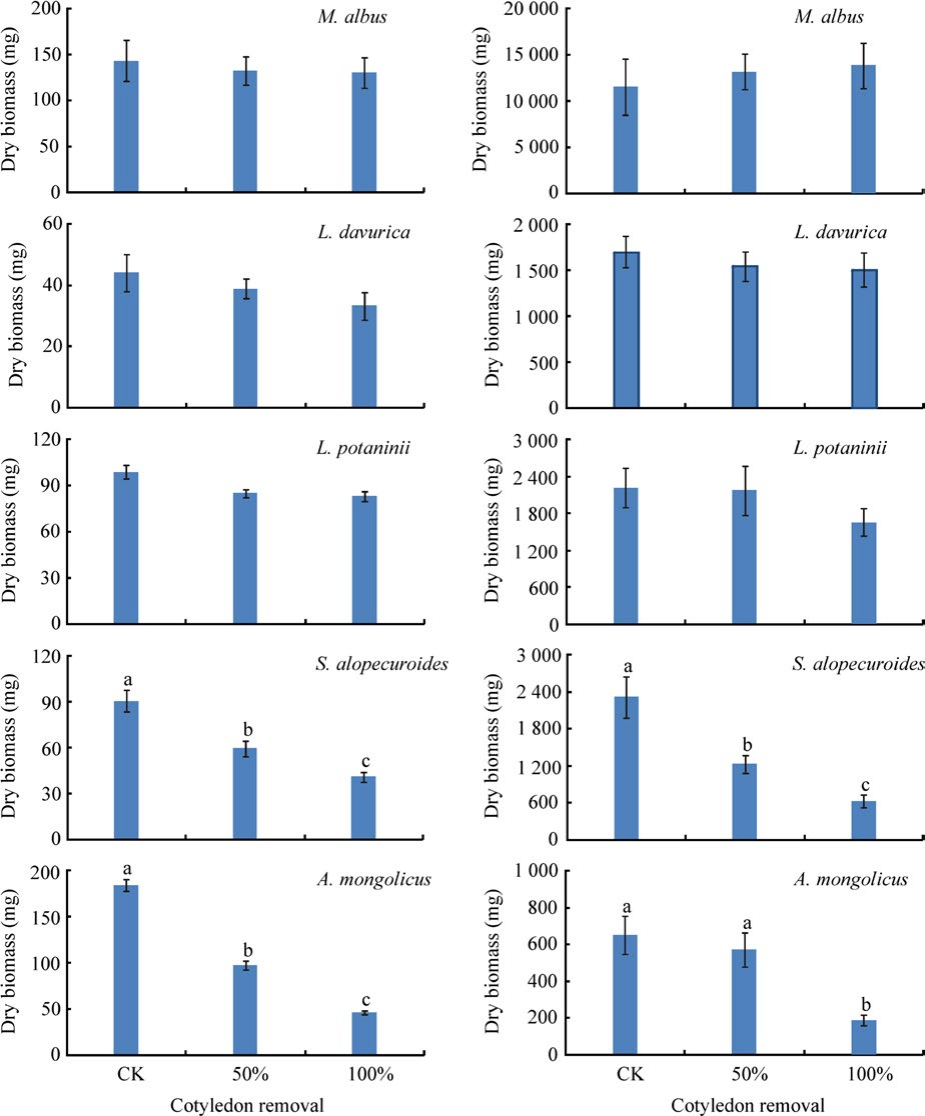
Seedling biomass (mean ± SE) of five legume species with cotyledon removal at 33 (left) and 70 days (right). Different letters within the species indicate significant differences at the 0.05 level based on Duncan's multiple range tests (*n* = 8/10)

Except for the interaction of cotyledon treatment (*CT*) and species (*S*), of *CT*,* S,* and sample time (*ST*), *S*,* CT*,* ST,* and their interactions showed significant effects on seedling biomass (Table 1). Cotyledon removal showed no effect on seedling biomass of *Lespedeza potaninii*,* L*. *dahurica,* and *Melilotus albus* regardless of time. In contrast, seedling biomass was significantly reduced by cotyledon removal in *Ammopiptanthus mongolicus* and *Sophora alopecuroides* both at 33 and 70 days after treatment, except for 50% cotyledon removal in *A. mongolicus* at 70 days (Figure 1).

**Table 1:** Three‐way ANOVA of the effects of species (*S*), sample time (*ST*), cotyledon removal treatment (*CT*), and their interactions on seedling biomass and relative growth rate of five legume species (*n* = 10)

Source of variation	*df*	*SS*	*MS*	*F*	*P*
Seedling biomass					
Species (*S*)	4	18.80341	4.70085	149.18	<.001
Cotyledon treatment(*CT*)	2	2.67126	1.33563	42.39	<.001
Sample time(S*T*)	1	122.1517	122.1517	3876.46	<.001
*S *× *CT*	8	2.62905	0.32863	10.43	<.001
*S *× *ST*	4	13.2086	3.30215	104.79	<.001
*CT *× *ST*	2	0.04481	0.0224	0.71	.492
*S *× *CT *× *ST*	8	0.28533	0.03567	1.13	.342
Relative growth rate					
Species (*S*)	4	0.409431	0.102358	384.68	<.001
Cotyledon treatment(*CT*)	2	0.004421	0.00221	8.31	<.001
Sample time(S*T*)	1	0.074075	0.074075	278.39	<.001
*S*×*CT*	8	0.005745	0.000718	2.7	.007
*S*×*ST*	4	0.047038	0.01176	44.2	<.001
*CT *× *ST*	2	0.003541	0.001771	6.65	.002
*S *× *CT *× *ST*	8	0.003301	0.000413	1.55	.141

**Figure 2:**
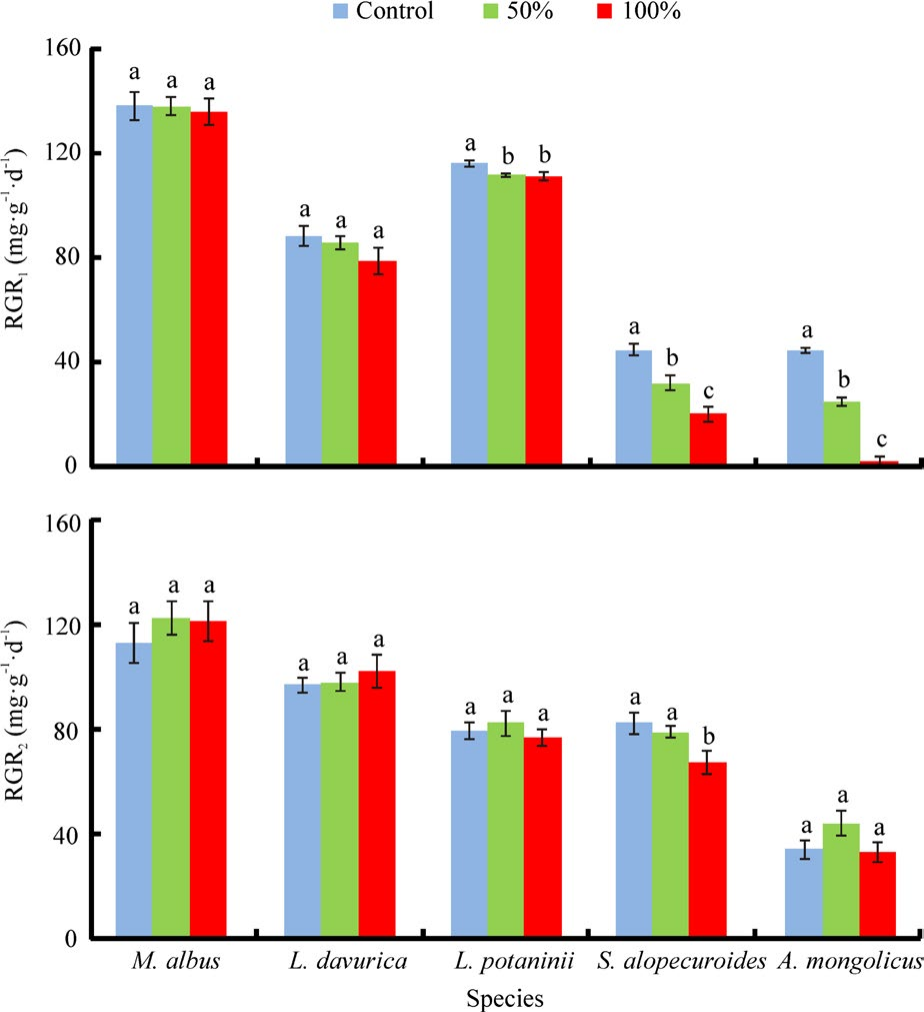
Effect of species and cotyledon removal on seedling relative growth rate (RGR) (mean ± SE) at different growth stages. RGR_1_ and RGR_2_ indicated relative growth rate between 28 May to 30 June 2014, and 30 June to 6 August 2014, respectively. Different letters within the species indicate significant differences at each growth stage based on Duncan's multiple range tests (*P *< .05, *n* = 8/10)

Except for the interaction of *S*,* CT,* and *ST*,* S*,* CT*,* ST,* and their interactions showed significant effects on seedling relative growth rate (RGR; Table 1). Seedling RGR in the first 33 days was significantly reduced by cotyledon removal in *L. potaninii*,* A. mongolicus* and *S. alopecuroides*, whereas no significant effect was observed in *L*. *dahurica* and *M. albus*. Meanwhile, RGR from 33 to 70 days was not reduced by cotyledon removal in any of the species, except for 100% cotyledon removal in *S. alopecuroides* (Figure 2).

### 3.2 Effect of cotyledon removal on seedling survival

Final survival of seedlings varied with the species and amount of cotyledon removal (Table 2). Generally, cotyledon removal had a significant effect on final seedling survival in *A. mongolicus* and *S. alopecuroides*, and a significant difference was observed between control and 100% but not 50% cotyledon removal treatment in both species. In contrast, cotyledon removal showed no effect on final seedling survival in *L. potaninii*,* L*. *dahurica,* and *M. albus*. All seedlings of *M. albus* died before winter regardless of treatment.

**Table 2:** Numbers of seedlings of five legume species surviving for 1, 2, and 12 months after cotyledon removal treatments. The numbers in parenthesis are final percentages of survival. Different letters within the species indicate significant difference at each growth stage based on chi‐square test (*P *< .05)

Species	Treatment	Initial no. seedlings number	Seedling no. after 1 month	Seedling no. after 2 months	Seedling no. after 12 months (%)
*M. albus*	Control	30	23	23	–
	50% cotyledon removal	30	21	21	–
	100% cotyledon removal	30	23	23	–
*L. davurica*	Control	30	15	14	14a (47)
	50% cotyledon removal	30	16	14	14a (47)
	100% cotyledon removal	30	14	13	13a (43)
*L. potaninii*	Control	30	29	27	27a (90)
	50% cotyledon removal	30	29	27	27a (90)
	100% cotyledon removal	30	30	28	28a (93)
*S. alopecuroides*	Control	30	27	25	20a (67)
	50% cotyledon removal	30	27	25	18ab (60)
	100% cotyledon removal	30	25	22	12b (40)
*A. mongolicus*	Control	30	23	23a	21a (70)
	50% cotyledon removal	30	24	21a	18a (60)
	100% cotyledon removal	30	18	9b	6b (20)

## 4 DISCUSSION

Our data clearly show that seedlings from species with small seeds were less affected by cotyledon removal than those from species with large seeds, regardless of seedling growth or their survival probability in the field. These results contradict the commonly held view that there is a positive relationship between seed size and the ability of seedlings to recover from herbivore damage (Armstrong & Westoby, 1993; Green & Juniper, 2004; Harms & Dalling, 1997). However, it is consistent with the results of Hanley and May (2006) who found that two species unaffected by cotyledon damage (*Cerastium holostoides* and *Senecio jacobaea*) were those with the smallest seeds among the nine species tested.

A possible interpretation for our results is the detrimental effect of cotyledon damage on plant size had disappeared by the time of harvest, given a higher RGR of seedlings from small than large seeds. Indeed, the RGRs of *L. potaninii*,* L*. *dahurica,* and *M. albus* were significantly higher than those of *S. alopecuroides* and *A. mongolicus* in the first 33 days of our study (Figure 2). Thus, seedlings from small seeds recovered more rapidly from cotyledon damage than those from large seeds. This also explains the differing effects of cotyledon removal on seedling growth of *A. mongolicus* in which seedling biomass was significantly reduced by 50% cotyledon removal at 33 days but not at 70 days. Our results are consistent with those of Hanley and May (2006) who showed that cotyledon removal from 7‐day‐old seedlings significantly reduced plant growth in six of nine species at 28 days (after cotyledon removal), whereas it did so in only two species at 100 days.

However, it is also worth noting that the results may have been influenced by at least one important reason. The species we used are from two different habitats, that is, cold desert and ruderal, and differences in adaptation capability and ecological characteristics may complicate the effects of seed size. For example, species from the cold desert may be more tolerate to drought compared to those from ruderal habitat, and thus less affected by harsh environment. *Lespedeza potaninii* and *L. dahurica* from the cold desert and ruderal habitats, respectively, had 93% and 43% final seedling survival, when both cotyledons were removed, although mass of individual seeds was 0.0023 g and 0.0022 g, respectively.

Seedling size at the initial harvest was ranked according to seed size: *A. mongolicus* > *S. alopecuroides* > *L. potaninii* > *L. dahurica* > *M. albus*. However, at 33 days, seedling size was *A. mongolicus* > *M. albus* > *L. potaninii* > *S. alopecuroides* > *L. dahurica*, and at 70 days, it was *M. albus* > *L. potaninii* > *L. dahurica* > *S. alopecuroides* > *A. mongolicus*. These results are consistent with our hypothesis that the initial advantage of large seedlings from large seeds is gradually overtaken by seedlings from small seeds having a higher RGR than those from large seeds. This conclusion is further supported by the correlation analysis which showed that seed mass was significantly positively related to seedling biomass at 33 days (*r* = .381, *P* = .006) but significantly negatively related to seedling biomass at 70 days (*r* = −.399, *P* = .011) (Appendix. 1). Moreover, this advantage in RGR of small seeds was further increased by cotyledon removal which significantly reduced RGR of seedlings from large seeds but not for those from small seeds at 33 days, suggesting seed reserves are more important for seedlings from large seeds than those from small seeds to maintain high RGR during early growth.

It previously has been emphasized that seedlings from large seeds are more tolerant of cotyledon removal than those from small seeds due to more storage food in large than in small seeds (Foster & Jason, 1985; Kitajima, 1996; Westoby, 1996). However, Lamont and Groom (2002) experimentally showed that cotyledons in *Hakea* spp. controlled seedling mass and morphology by supplying mineral nutrients rather than organic compounds. Further, cotyledon removal had no effect on seedling growth of *Hakea* spp. when additional nutrients were supplied, suggesting the function of cotyledon reserves may strongly depend on seedling growth conditions. Milberg, Pérez‐Fernández, and Lamont (1998) have shown that seedlings from species with large seeds seemed to be unable to use extra nutrients supplied during early growth, whereas those from small‐seeded species responded strongly to nutrient availability. Consistent with this, RGR were greatly reduced by cotyledon removal in *S. alopecuroides* and *A. mongolicus* in the first 33 days of our study, but no detrimental effect was observed in *L. potaninii*,* L*. *dahurica,* and *M. albus*. These results support our hypothesis that the initial growth of seedlings from large seeds is more depended on seed reserves in particularly at early seedling stage than that of seedlings from small seeds. A possible reason is that seedlings from small‐seeded species may have a stronger capability to use external nutrients than those from large‐seeded species during early growth, thus compensating for the loss of seed reserves.

Slot, Palow, and Kitajima (2013) found that N and P deficiencies of *Leucaena leucocephala* seedlings became significant at 16 and 31 days, respectively, when they were incubated in a nutrient‐free solution. At this time (>16 days), seedlings already have a developed root system, and thus, they can utilize soil nutrients at least in part. In our study, the soil available nitrogen, phosphate, and potassium was 12.1, 25.5, and 113.3 mg/kg, respectively; thus, some nutrients for seedling growth may have come from the soil, thereby reducing the dependence of seedlings on cotyledon reserves.

As growth conditions in our study were less stressful (absence of competition, abundant soil nutrients) than those of true field conditions, they may have been more beneficial to the small than the large‐seeded species (Seiwa & Kikuzawa, 1991, 1996). It is likely that competition with established vegetation would amplify even minor reductions in individual size that occur during the early part of the life of the plant (Hanley & May, 2006). Thus, the function of cotyledons needs to be tested under a range of conditions.

Death of seedlings without cotyledon removal mainly occurred in the first 33 days after seedling emergence for all our tested species. A possible reason why a high percentage of the seedlings died is that rainfall from 0 to 33 days was only 57.1 mm, whereas it was 164.2 mm from 33 to 70 days. Moreover, newly emerged seedlings are less tolerant to drought stress than older ones with an established root system. However, cotyledon removal obviously changed this pattern of more death in young than old seedlings, especially for *S. alopecuroides* and *A. mongolicus* in which seedling death greatly increased during winter. These results imply that reduced seedling growth due to cotyledon removal may have caused seedlings to fail to overwinter.

Further, our results showed no correlation between seed mass and seedling survival regardless of time when seeds remained intact. Moles and Westoby (2004) found a nonsignificant positive relationship between seed mass and early seedling survival for 40 species from temperate grasslands. No relationship was found between seed mass and the proportion of seedlings surviving to reproductive maturity for 19 species from 12 families. However, their study found that significant positive relationship between seed mass and early seedling survival for 63 species from temperate forests or shrub lands, suggesting that the advantage of large seeds is habitat dependent. Moreover, our study showed that survival at 2 and 12 months was higher for seedlings from small than from large seeds when both cotyledons were removed, suggesting that food reserves are more important for seedlings from large than from small seeds. Thus, seedling survival in the field involves seed mass, insect predation (cotyledon removal), environmental conditions, and life history of the species, including seedling growth rates. However, it should be noted that the number of species used in our study was limited, and thus, a robust conclusion about the relationship between seed mass and seedling growth and survival needs a further work involving additional species.

Many factors such as species ecological characteristics, growth conditions, and growth stage may be important in dictating the observed response of plants to cotyledon damage as discussed above. Nevertheless, our study clearly showed that seedlings from small seeds are more tolerate of cotyledon damage than those from large seeds in semi‐field conditions, suggesting that seed energy reserves are more important for the early growth of seedlings from large than small seeds. The overall effect of cotyledon removal on growth and survival varies with seed size (i.e., energy reserves) with seedlings from small seeds being less sensitive than those from large seeds under semi‐field conditions.

## CONFLICT OF INTEREST

None declared.

## APPENDIX 1

### 1.1

Correlation between seed mass, seedling biomass, and seedling relative growth rate at33 (upper) and 70 (lower) days, in the cotyledon damage experiment (*n* = 8/10).
